# Roles of Setbp1 in developmental hematopoiesis and acute myeloid leukemia

**DOI:** 10.1016/j.gendis.2023.101195

**Published:** 2023-12-13

**Authors:** Fei Ao, Fan Chen, Minhong Lv, Yuming Cao, Jinfeng Xu, Jingbo Xu, Anskar Yu-Hung Leung, Qiwen Yuan, Li Wang, Bailiang He

**Affiliations:** a Guangdong Provincial Engineering Research Center of Molecular Imaging, The Fifth Affiliated Hospital, Sun Yat-sen University, Zhuhai, Guangdong 519000, China; b Department of Gynecology and Obstetrics, Perinatal Medical Center, The Fifth Affiliated Hospital, Sun Yat-sen University, Zhuhai, Guangdong 519000, China; c Department of Hematology, The Fifth Affiliated Hospital, Sun Yat-sen University, Zhuhai, Guangdong 519000, China; d Division of Haematology, Department of Medicine, School of Clinical Medicine, Li Ka Shing Faculty of Medicine, The University of Hong Kong, Hong Kong 999077, China

Gain-of-function somatic mutations of SET binding protein 1 (*SETBP1*) result in the accumulation of SETBP protein and are detected in 17% of secondary acute myeloid leukemia (AML) patients.^1^ In fact, high expression of *SETBP1* also drives adverse outcomes in human AML. However, the roles of *SETBP1* during developmental hematopoiesis and AML progression are still not fully understood.

Here we first sought to investigate the functions of *SETBP1* in developmental hematopoiesis. *SETBP1* is highly expressed in hematopoietic stem cells compared with other progenitor cells including common myeloid progenitor, granulocyte-monocyte progenitor, and megakaryocytic-erythroid progenitor cells (Fig. 1A). *SETBP1* knockout is embryonic lethal in mice, precluding detailed investigations of its functions. We and others reveal that zebrafish is a versatile animal model to study hematopoiesis and leukemogenesis.^2^ Mammalian and zebrafish Setbp1 proteins are evolutionarily conserved based on syntenic neighboring gene analysis and multiple sequence alignment (Fig. 1B, C). *SETBP1* is expressed in normal tissues in humans and in zebrafish embryos (Fig. S1, 2). A morpholino (MO hereafter, Table S1) specifically blocking the translation of *setbp1* was designed, and microinjected into one-cell stage embryos (*setbp1* morphant hereafter) (Fig. 1D, E). Setbp1-MO was effective in blocking the translation of an artificially generated *5′-UTR-setbp1-egfp* chimeric gene, confirming its high knockdown efficiency *in vivo* (Fig. S3). *cmyb*^+^ hematopoietic stem and progenitor cells (Fig. 1F–i), *pu.1*^+^ myeloid progenitor cells (Fig. 1F–ii), and *mpo*^+^ neutrophils (Fig. 1F–iii) were significantly reduced in the posterior blood island from *setbp1* morphant, while the *gata1*^+^ erythrocytes were increased (Fig. 1F–iv). The perturbation of hematopoiesis in *setbp1* morphant was not due to the defects of blood vessels as their dorsal aorta, dorsal vein, and intersegmental vessels are intact (Fig. S4). These data indicate that *setbp1* is required for developmental hematopoiesis in the zebrafish model.

**Figure 1:**
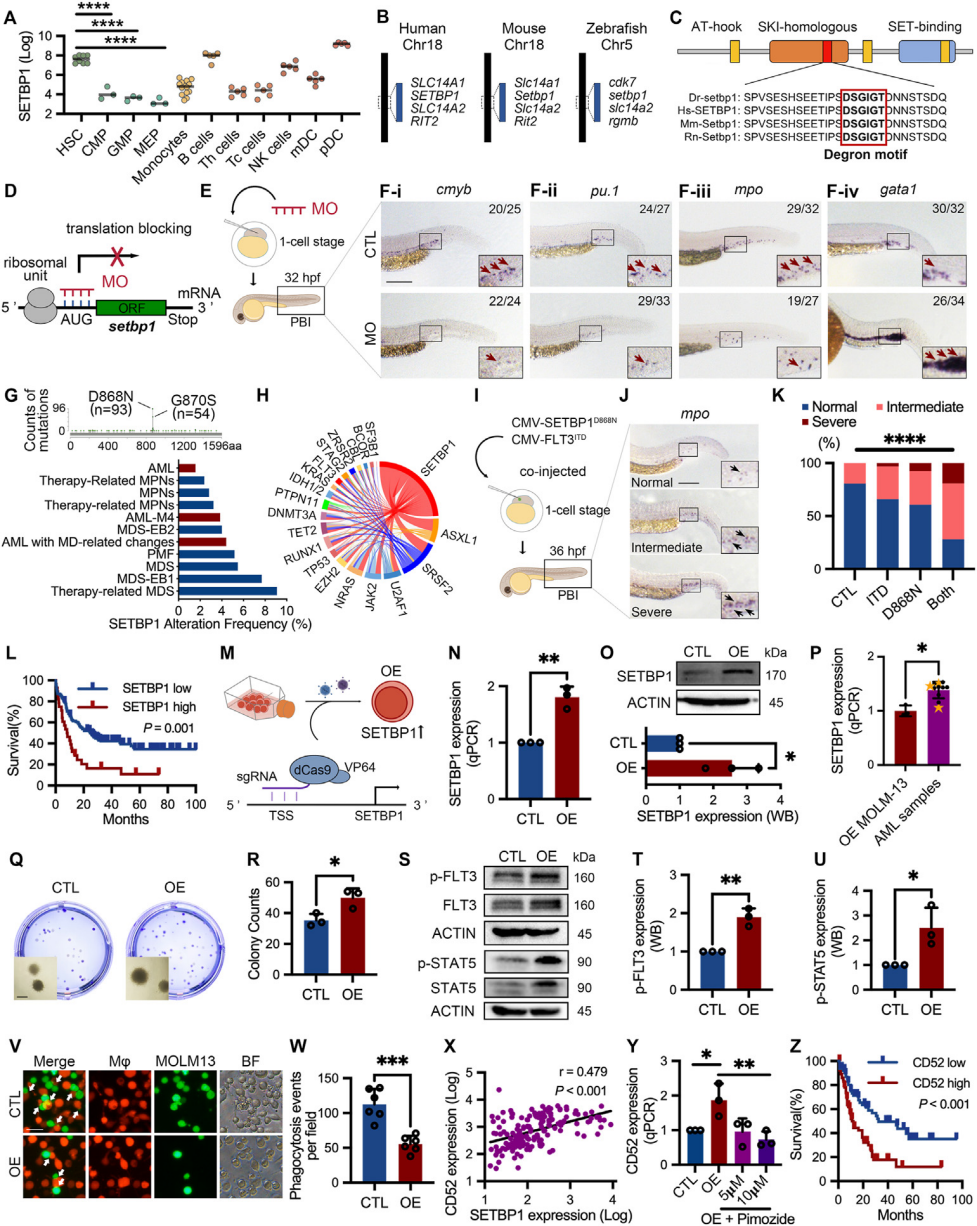
Roles of Setbp1 in hematopoiesis and AML. **(A)** The expression of *SETBP1* in different blood cell populations was analyzed by the BloodSpot program. HSC, hematopoietic stem cell; CMP, common myeloid progenitor; GMP, granulocyte monocyte progenitor; MEP, megakaryocyte-erythroid progenitor; Monocytes, CD14^+^ monocytes; B cells, CD19^+^ B cells; Th cells, CD4^+^ T-helper cells; Tc cells, CD8^+^ cytotoxic T cells; NK cells, CD56^+^ natural killer cells; mDC, CD11c^+^ myeloid dendritic cells; pDC, CD123^+^ plasmacytoid dendritic cells. **(B, C)** Syntenic neighboring gene analysis (B) and multiple sequence alignment (C) detecting the conservation of SETBP1 from mammalian and zebrafish. Dr, *Danio rerio*; Hs, *Homo sapiens*; Mm, *Mus musculus*; Rn, *Rattus norvegicus*. **(D, E)** Schematic diagram depicting the design of antisense morpholino (MO) (D) and *setbp1* knockdown by MO through microinjection into one-cell stage zebrafish embryos (E). Scramble morpholino was used as control (CTL). **(F)** Whole-mount *in situ* hybridization assay detecting the expression of *cmyb*, *pu.1*, *mpo*, and *gata1* in the posterior blood island (PBI) region of zebrafish embryos. Scale bar = 200 μm. **(G, H)** Alteration frequency of *SETBP1* (G) and co-occurred genes (H) in myeloid malignancies were revealed using the cBioPortal program. MPN, myeloproliferative neoplasms; MDS, myelodysplastic syndromes; PMF, primary myelofibrosis. **(I–K)** Schematic diagram depicting the co-expression of *SETBP1*^D868N^ and *FLT3*^ITD^ in zebrafish embryos through microinjection of plasmid DNA at one-cell stage (I). Embryos were classified into three categories (normal, intermediated, and severe) (J) based on the levels of *mpo*^+^ myeloid cells, and percentages from different groups were quantified (K). Scale bar = 200 μm. **(L, M)** Overall survival analysis (L) (log-rank test) of patients from TCGA-LAML database based on differential expression of *SETBP1* (M). **(N–P)** Schematic diagram depicting the overexpression of *SETBP1* (OE) in *FLT3*^ITD^-mutated MOLM-13 cells by CRISPRa-mediated transcriptional activation (N). MOLM-13 cells transduced with scramble sgRNA were used as control (CTL). The overexpression of *SETBP1* was confirmed by RT-qPCR (O) and western blotting (P), respectively. The transcriptional levels of *SETBP1* in CRISPRa-transduced MOLM-13 cells and AML patient samples were detected by RT-qPCR and compared after normalizing to their corresponding GAPDH (P). **(Q, R)** The clonogenicity of MOLM-13 cells (CTL *vs*. OE) was measured by colony-forming unit assay *in vitro*. Scale bar = 50 μm. **(S–U)** Western blotting detecting FLT3-related signaling in MOLM-13 cells (CTL *vs*. OE) (S). The intensities of bands from p-FLT3 and p-STAT5 were quantified by ImageJ (T, U). **(V, W)** Detection of phagocytosis after co-culturing of EGFP^+^ MOLM-13 cells and mCherry^+^ macrophages derived from THP-1 monocytic cells (V). Phagocytosis events per field were recorded and quantified (W). Scale bar = 10 μm. **(X)** The correlation between *SETBP1* and *CD52* expression was analyzed (Pearson test) in patients from the TCGA-LAML database. **(Y)** Transcription of *CD52* was detected in MOLM-13 cells (CTL, OE, and OE treated with STAT5 inhibitor Pimozide). **(Z)** Overall survival analysis (log-rank test) of patients from TCGA-LAML database based on *CD52* expression.

We then sought to study the pathogenic roles of SETBP1 during leukemogenesis. Consistent with previous observations,^1^ *SETBP1* mutations (D868N and G870S) are commonly detected in a variety of myeloid malignancies including AML (Fig. 1G), and co-occurred with genetic alterations involved in spliceosome (*SRSF2*, *U2AF1*, *SF3B1*), epigenetics (*ASXL1*, *EZH2*, *TP53*, *TET2*, *DNMT3A*, *IDH1/2*), kinase signaling (*JAK2*, *NRAS*, *PTPN11*, *KRAS*, *FLT3*) (Fig. 1H). We previously demonstrated that the cooperative effects of leukemic oncogenes could be readily tested in zebrafish embryos through plasmid microinjection.^2^ Accordingly, the full-length sequence of *SETBP1*^D868N^ and *FLT3*^ITD^ mutations were cloned into a vector to generate CMV-*SETBP1*^D868N^-T2A-EGFP and CMV-*FLT3*^ITD^-T2A-EGFP recombinant plasmid DNA, respectively. The expression of *SETBP1*^D868N^ and *FLT3*^ITD^ mutations was confirmed by the detection of *EGFP* expression in the embryos after plasmid microinjection (data not shown). Consistently, while overexpression of low dosage of human *SETBP1*^D868N^ and *FLT3*^ITD^ mutations only induced mild expansion of *mpo*^+^ myeloid cells respectively (Fig. 1I), co-overexpression of them elicited synergistic effects to induce more severe myeloid expansion in zebrafish embryos (Fig. 1J, K). These data suggest that overexpression of *SETBP1* cooperates with *FLT3*^ITD^ to promote myeloid expansions in the zebrafish model.

High expression of SETBP1 is associated with adverse prognosis in human AML (Fig. 1L). Though *SETBP1* was reported to cooperate with *FLT3*^ITD^ mutation to drive AML in mice,^3^ its roles in human *FLT3*^ITD^-mutated AML cells are still elusive. Therefore, we then overexpress *SETBP1* in FLT3-ITD-mutated MOLM-13 cells which show low *SETBP1* levels comparing to those of AML samples (Fig. S5; Table S3). Transcriptional up-regulation of *SETBP1* in MOLM-13 cells was achieved by using clustered regularly interspaced short palindromic repeats (CRISPR) activation (CRISPRa) system, in which a sgRNA-guided nuclease deficient CRISPR-associated protein 9 (dCas9) is fused with VP64 transcription activator (Fig. 1M). Compared with the scramble sgRNA, the introduction of sgRNA targeting the transcriptional start site of the *SETBP1* gene results in about a two-fold increase of *SETBP1* transcription and protein expression (Fig. 1N, O) which are comparable with those observed in AML patient samples (Fig. 1P). Functionally, CRISPRa-mediated up-regulation of *SETBP1* promotes the clonogenicity of MOLM-13 cells *in vitro* (Fig. 1Q, R). As the growth of MOLM-13 is dependent on the activity of FLT3 and its downstream STAT5, PI3K/AKT, MAPK/ERK signaling, FLT3-related signaling molecules were then detected by western blotting after SETBP1 overexpression. Unexpectedly, SETBP1 overexpression results in increased phosphorylation of FLT3 (Fig. 1S, T) and STAT5 (Fig. 1U), but not PI3K/AKT and MAPK/ERK signaling (Fig. S6). These data indicate that *SETBP1* overexpression activates STAT5 to promote the aggressiveness of *FLT3*^ITD^-mutated AML cells.

The above observations prompted us to further delineate the molecular mechanism(s) by which high expression of *SETBP1* is implicated. Gene set enrichment analysis reveals that the *SETBP1*-associated genes are associated with KEGG pathways of “adaptive immune response”, “leukocyte cell–cell adhesion”, “leukocyte proliferation”, “STAT cascade”, “interleukin-10 production”, *etc* (Fig. S7A). Unexpectedly, high expression of *SETBP1* in AML is associated with increased frequencies of M2-like macrophages (Fig. S7B) which are one of the major producers of IL-10. Consistently, expression of *SETBP1* is significantly correlated with IL-10 receptor IL10RA in AML cases (Fig. S7C), suggesting the potential interplay between the *SETBP1*^high^ AML cells and macrophages. We then performed co-culture experiments to test this hypothesis. Unexpectedly, *SETBP1*-overexpressed MOLM-13 cells significantly impaired the phagocytic activities of THP-1 monocyte-derived macrophages *in vitro* based on fluorescent imaging (Fig. 1V, W) and flow cytometry analysis (Figs. S8A–C). Reduced phagocytic activities were also demonstrated using umbilical cord blood mononuclear cell-derived macrophages (Fig. S8D, E). The most well-described innate immune checkpoints are the “don't eat me” signals, including the CD47/SIRPα, PD-1/PD-L1, CD52/SIGLEC-10,^4^ CD24/SIGLEC-10, and HLA-G/LILRBs axis, *etc*. Expression of *CD52* (Fig. 1X) and *PD-L1* (Fig. S9A), but not *CD24*, *HLA-G*, and *CD47* (Fig. S9B–D), are significantly correlated with *SETBP1* in AML patients. Importantly, *CD52* (Fig. 1Y), but not *PD-L1* (Fig. S9E), is significantly up-regulated in *SETBP1*-overexpressed MOLM-13 cells. Pharmacologically, the increase of *CD52* in *SETBP1*-overexpressed MOLM-13 cells is reduced upon the treatment of STAT5 inhibitor pimozide (Fig. 1Y). In fact, *CD52* is a poor prognostic factor in AML (Fig. 1Z) and potentially regulated by STAT5 as predicted by computational methods such as HOMER and hTFtarget (Table S4, 5). These data indicate that overexpression of *SETBP1* in *FLT3*^ITD^-mutated AML cells may up-regulate CD52 to reduce the phagocytic activities of leukemia-associated macrophages.

When our initial submission was under review, a comprehensive investigation from Atsushi Tanaka and colleagues indicates that *SETBP1* is dispensable for normal and malignant hematopoiesis based on the elegant work in mouse model.^5^ Atsushi Tanaka et al demonstrate that *SETBP1* depletion in normal hematopoiesis minimally alters self-renewal, differentiation, or reconstitution in a mouse model. We propose that the different hematopoietic phenotypes in *setbp1* knockdown zebrafish and *Setbp1* KO mice may be attributed to several reasons, such as the potential non-cell autonomous effects of *Setbp1* in hematopoiesis, potential roles of *Setbp1* in the initiation and specification of hematopoietic stem and progenitor cells, potential genetic compensation in *Setbp1* KO hematopoietic stem cells, as well as the inherent diversity of zebrafish and mouse models. Though *SETBP1* is dispensable for the development or maintenance of AML as reported, we show that up-regulation of endogenous *SETBP1* with a physiologically relevant level in *FLT3*^ITD^-mutated MOLM-13 cells by CRISPR activation (but not retrovirus transduction) promote the aggressiveness of *FLT3*^ITD^ AML cells via activation of FLT3/STAT5 signaling.

Taken together, our data indicate that *Setbp1* is required for embryonic hematopoiesis in the zebrafish model. Overexpression of *SETBP1* promotes the aggressiveness of *FLT3*^ITD^-mutated AML cells by activating STAT5. Inhibition of STAT5 may represent novel therapeutics in *SETBP1*^high^/*FLT3*^ITD^ AML patients.

## Ethics declaration

All animal studies have been approved by the Ethical Committee at The Fifth Affiliated Hospital of Sun Yat-sen University. Informed consent was obtained from all subjects and the human studies were approved by the Institutional Review Boards from The Fifth Affiliated Hospital of Sun Yat-sen University. All experiments conformed to the principles set out in the WMA Declaration of Helsinki and the Department of Health and Human Services Belmont Report.

## Author contributions

F.A., F.C., Q.W.Y., L.W., and B.L.H. conceived the project and designed the experiments. F.A., F.C., M.H.L., Y.M.C., J.F.X., and B.L.H. carried out the experiments and analyzed the data. J.B.X., A.Y.H.L., L.W., and B.L.H. analyzed the clinical data. F.A., F.C., Q.W.Y., L.W., and B.L.H. reviewed, edited, and revised the manuscript. B.L.H. directed and supervised the project. All authors discussed the results and approved the submission of the manuscript.

## Conflict of interests

The authors declare that they have no competing interests.

## Funding

This project was supported by grants from the 10.13039/501100001809National Natural Science Foundation of China (No. 32000569) and the 10.13039/501100021171Basic and Applied Basic Research Foundation of Guangdong Province, China (No. 2019A1515110281).

## Data availability

The datasets used and/or analyzed during the current study are available from the corresponding author upon reasonable request.
