# Supplementary material for: Roles of Setbp1 in developmental hematopoiesis and acute myeloid leukemia
Source: Genes Dis. 2023 Dec 13;11(6):101195. doi: 10.1016/j.gendis.2023.101195 (PMC11299574; doi:10.1016/j.gendis.2023.101195)
Supplement: Multimedia component 1 [file mmc1.pdf]

Roles of Setbp1 in developmental hematopoiesis and acute myeloid leukemia

Running title: Roles of Setbp1 in developmental hematopoiesis and AML

Fei Ao [a,b,#], Fan Chen [a,#], Min-Hong Lv [a,#], Yu-Ming Cao [b], Jin-Feng Xu [b], Jing-Bo Xu [c], Anskar Yu-Hung Leung [d], Qi-Wen Yuan [b,*], Li Wang [b,*], Bai-Liang He (何柏亮) [a,*]

[a] Guangdong Provincial Engineering Research Center of Molecular Imaging, The Fifth Affiliated Hospital, Sun Yat-sen University, Zhuhai, Guangdong, 519000, P.R. China.

[b] Department of Gynecology and Obstetrics, Perinatal Medical Center, The Fifth Affiliated Hospital, Sun Yat-sen University, Zhuhai, Guangdong, 519000, P.R. China.

[c] Department of Hematology, The Fifth Affiliated Hospital, Sun Yat-sen University, Zhuhai, Guangdong, 519000, P.R. China.

[d] Division of Haematology, Department of Medicine, Li Ka Shing Faculty of Medicine, The University of Hong Kong, Pokfulam, Hong Kong, 999077, P.R. China.

[#]These authors contributed equally to this work.

***Correspondence:**

Bai-Liang He (何柏亮), Ph.D.

Guangdong Provincial Engineering Research Center of Molecular Imaging, The Fifth Affiliated Hospital, Sun Yat-sen University, Zhuhai, Guangdong, 519000, P.R. China.

E-mail address: **hebliang@mail.sysu.edu.cn**

Li Wang, M.D. & Ph.D.

Department of Gynecology and Obstetrics, Perinatal Medical Center, The Fifth Affiliated Hospital, Sun Yat-sen University, Zhuhai, Guangdong, 519000, P.R. China.

E-mail address: wangli223@mail.sysu.edu.cn

Qi-Wen Yuan, M.D.

Department of Gynecology and Obstetrics, Perinatal Medical Center, The Fifth Affiliated Hospital, Sun Yat-sen University, Zhuhai, Guangdong, 519000, P.R. China.

E-mail address: yuanqw@mail.sysu.edu.cn

Competing interests: The authors declare that they have no competing interests.

Key words: SETBP1, Zebrafish, Hematopoiesis, Acute Myeloid Leukemia, $FLT3^{ITD}$, STAT5, CD52

Word counts: 1200

Main Figure: 1

Supplemental files: 9 supplemental figures, 5 supplemental tables

Supplemental files

Materials and Methods

Bioinformatics analysis

The relative expression of *SETBP1* in different human tissues (HPA RNA-seq normal tissues)[1] and normal hematopoietic cells (HemaExplorer, GSE17054, GSE19599, and GSE11864) was analyzed using the National Center for Biotechnology Information (NCBI) gene portal and BloodSpot program, respectively. SETBP1 protein sequences from zebrafish (*Danio rerio, Accession: XP_005165732.1*), human (*Homo sapiens, Accession: NP_001366070.1*), mouse (*Mus musculus, Accession: NP_444329.2*), and rat (*Rattus norvegicus, Accession: XP_038953130.1*) were retrieved from NCBI and used for multiple sequence alignment by Clustal Omega program with default settings. The genetic alterations of *SETBP1* in myeloid malignancies were analyzed using the cBioportal program. The overall survival of AML patients (TCGA-LAML database) based on *SETBP1* or *CD52* transcription was analyzed by the OncoLnc program. The correlation between *SETBP1* and *CD52* transcription in AML patients (TCGA-LAML database) was analyzed by the LinkedOmics program. The transcription factor binding sites on human CD52 promoter were predicted by HOMER and hTFtarget portal, respectively. The datasets analyzed in the present study are publicly available in NCBI gene portal (https://www.ncbi.nlm.nih.gov/gene/26040), BloodSpot (https://servers.binf.ku.dk/bloodspot/),[2] NCBI (https://www.ncbi.nlm.nih.gov/), Clustal Omega (https://www.ebi.ac.uk/Tools/msa/clustalo/), cBioportal (https://www.cbioportal.org/),[3] OncoLnc (https://www.oncolnc.org/), LinkedOmics

(https://www.linkedomics.org/),[4] SangerBox (https:// http://sangerbox.com/), HOMER (http://homer.ucsd.edu/homer/),[5] and hTFtarget (http://bioinfo.life.hust.edu.cn/hTFtarget#!/).[6]

Zebrafish husbandry, morpholino, and plasmid microinjection

Wild-type AB and Tg(fli1:egfp) zebrafish lines (China Zebrafish Resource Center, Wuhan, China) were maintained under standard conditions (28.5℃, 14 hours with light) in an automatically circulating system (Haisheng, Shanghai, China). Zebrafish embryos were collected by natural spawning, kept in an E3 culture medium (5 mM NaCl, 0.17 mM KCl, 0.33 mM $CaCl_2$, and 0.33mM $MgSO_4$ in ddH_2O with methylene blue), and staged as hour post-fertilization (hpf) and day post-fertilization (dpf) as described.[7, 8] A zebrafish *setbp1*-specific translation block morpholino (setbp1-MO, Table S3) was designed (Gene tools, Oregon, USA) and microinjected into one-cell stage embryos (4 ng per embryo) using Warner PLI-100A Pico-Injector (Harvard Apparatus, Massachusetts, USA). A standard scramble sequence morpholino (Gene tools, Oregon, USA) was used as control (CTL-MO, Table S3). For plasmid microinjection, the full length of the human *FLT3*^{ITD} sequence was PCR amplified from MOLM-13 cells and then subcloned into pEGFP-N3 plasmid as previously described.[9] Full-length human wild-type *SETBP1* sequence was cloned (IGE Biotechnology, Guangzhou, China) into pEGFP-N3 plasmid (pCMV-*SETBP1*-T2A-*EGFP*). Site-directed mutagenesis was performed (IGE Biotechnology, Guangzhou, China) to generate *SETBP1*^{D868N} point mutation (pCMV- *SETBP1*^{D868N}-T2A-*EGFP*). Plasmid was

extracted (EndoFree Mini Plasmid Kit II, TIANGEN BIOTECH, Beijing, China) from transformed Escherichia coli (DH5α, TIANGEN BIOTECH, Beijing, China) and microinjected into one-cell stage zebrafish embryos (25 pg per embryo) using Warner PLI-100A Pico-Injector (Harvard Apparatus, Massachusetts, USA). Embryos were kept in an E3 medium and collected for downstream analysis at defined stages. Embryos with abnormal developmental delay or severe deformity (less than 5%) were excluded from the analysis.

Whole-mount in situ hybridization

Total RNA was isolated (NucleoZol, MACHEREY-NAGEL, Germany) from 1dpf wild-type AB zebrafish embryos, and reverse transcribed into first strand cDNA (HiScript III RT SuperMix, Vazyme, Nanjing, China). The cDNA was used as a PCR template to amplify (2X Pro Taq Master Mix, Accurate Biology, Guangzhou, China) the partial sequence of zebrafish *setbp1* (Primers see Table. S3). The PCR product was cloned into a pGEM-T-easy vector (Promega, Beijing, China) and verified by bidirectional Sanger sequencing (IGE Biotechnology, Guangzhou, China). The vector was then linearized and used as a DNA template to generate the antisense *setbp1* RNA probe *in vitro* (DIG RNA Labeling Kit, Roche, Germany). The whole-mount *in situ* hybridization (WISH) assay of *setbp1* was performed as previously described.[10, 11] Briefly, zebrafish embryos were collected and fixed with 4% (wt/vol) paraformaldehyde (PFA, Biosharp, Hefei, China) in 1 x PBS at 4°C overnight and dehydrated in 100 % ethanol in -20°C for more than 4 hours. On day-1, embryos were hybridized in PHB+ buffer [50% formamide, 5 x SSC (NaCl 43.825 g, Citric acid trisodium salt 22.05 g, 1L ddH$_2$O), 50 µg/mL heparin, 500 µg/mL yeast rRNA, 5 mM EDTA, 0.1% Tween 20, 0.92 mM citric acid, pH 6.0, with 1 ng/µL DIG-labeled antisense *setbp1* RNA probe] at 65°C

overnight. On day-2, embryos were washed in serial SSC buffers and then incubated with AP-conjugated anti-DIG antibody (1:5000 in PBST with 5% lamb serum, Roche, Germany) at 4°C overnight. On day-3, embryos were washed in PBST buffer five times and incubated in NBT/BCIP substrate (Roche, Germany) at room temperature for 1-2 hours. The signaling was developed, visualized, and captured by Nikon SMZ800 microscopy mounted with a Nikon Digital Sight DS-Qi2 camera.

Cell processing and drug treatment

MOLM-13 and THP-1 cell lines are maintained and sub-cultured according to the supplier's protocol (ATCC or DSMZ) as described previously.[8, 12] Briefly, leukemia cell lines were maintained at 37℃ with 5% CO2 in RPMI medium (Solarbio, Beijing, China) supplemented with 10% FBS (Gibco, Thermo Fisher Scientific) and penicillin & streptomycin antibiotics (HyClone, Thermo Fisher Scientific). All cell lines were recently authenticated and confirmed as mycoplasma-free. Mononuclear cells (MNCs) were isolated by Ficoll-Paque plus (HyClone, Thermo Fisher Scientific) from umbilical cord blood samples, and preserved in liquid nitrogen until used. The anti-leukemic effects of candidate drugs were tested in leukemia cell lines as previously described.[12] Briefly, leukemia cells (0.5 x 10^6/mL, 3 mL) were plated in 6-well plates in triplicate and treated with defined concentrations of STAT5 inhibitor pimozide for 48 hours. An equal amount of DMSO was used as vehicle control.

CRISPR activation-mediated *SETBP1* overexpression

Transcriptional activation of endogenous *SETBP1* in MOLM-13 cells was achieved by the CRISPR activation system as previously described.[13] Lentivirus transduction was performed by spinfection (350g for 90 minutes) as previously described.[8, 12, 14] Briefly, MOLM-13 cells were transduced with lentivirus packaged from 3rd generation lentiviral vector (lenti-dCas-VP64_Blast) encoding for the dCas9-VP64 (transcriptional activator) with 2A Blasticidin resistance marker. Three days post-transduction, MOLM-13$^{dCas9\text{-}VP64}$ cells were enriched by Blasticidin (7 μg/mL) selection for 3 days. Subsequently, MOLM-13$^{dCas9\text{-}VP64}$ cells transduced with lentivirus encoding for a sgRNA (OE group) specifically targeting the transcription start site (TSS) of human *SETBP1* (Table S3, Tsingke Biotechnology, Beijing, China) with EGFP selection marker. A scramble sgRNA (Table S3, Tsingke Biotechnology, Beijing, China) was used as a control (CTL group). Three days post-transduction, scramble sgRNA or *SETBP1*-sgRNA-transduced MOLM-13$^{dCas9\text{-}VP64}$ cells were sorted by FACS based on EGFP expression (FACSaria FUSION Flow Cytometers, BD Biosciences). Lenti dCAS-VP64_Blast (Addgene plasmid # 61425) was a gift from Feng Zhang's lab.

Real-time quantitative PCR

Real-time quantitative PCR (RT-qPCR) was performed as previously described.[8] Total RNA was extracted (NucleoZol, MACHEREY-NAGEL, Germany) from MOLM-13 cells and reversely transcribed (HiScript III RT SuperMix, Vazyme, Nanjing, China) into first strand cDNA. Real-time quantitative PCR (RT-qPCR) was performed using SYBR Green qPCR Master Mix (Vazyme, Nanjing, China) by StepOnePlus Real-Time PCR System (QuantStudio 7 Flex, ABI). *GAPDH* was used as an internal control (Table.

S1). The relative expression of candidate genes was calculated using the $2^{-\triangle\triangle Ct}$ method.

Western Blotting

Western Blotting was performed as previously described.[14] MOLM-13 cells were collected, washed with 1x PBS twice, and lysed with lysis buffer (Cell lysis buffer for Western and IP, Beyotime, Shanghai, China) supplement with 1x protease and phosphatase inhibitors (Beyotime, Shanghai, China) on ice for 20 min. Lysates were centrifuged (13,300 g for 15 min at 4℃) and the supernatants were collected. Equal amounts of protein were separated by 10% SDS-PAGE gel, transferred to PVDF membranes, blocked with 5% milk in TBST, and then incubated with primary antibodies (Table S2) overnight at 4℃. After washing in TBST three times, the membranes were incubated in HRP-conjugated secondary antibody (Beyotime, Shanghai, China) at room temperature for 1 hour. After washing in TBST three times, the signals were detected by ECL (Immobilon Western HRP substrate, Merck Millipore, USA) and recorded by (iBright Imaging Systems, ThermoFisher). β-ACTIN was detected and used as a loading control. The band intensity was analyzed by ImageJ software and the relative protein expression was calculated after normalizing with the corresponding β-ACTIN.

Prediction of transcription factor binding sites on CD52 promoter

The transcription factor binding sites on human CD52 promoter were predicted by using computational methods such as HOMER (Hypergeometric Optimization of Motif Enrichment, v4.11)[5] as well as hTFtarget portal.[6] Briefly, the promoter sequence of human CD52 (-2000bp to +100bp relative to TSS) was retrieved and used as input for

the prediction of potential transcription factor binding sites. For HOMER analysis, findMotifs.pl program (http://homer.ucsd.edu/homer/microarray/index.html) was used to search the transcription factor binding motifs of length 9 to 12 bp form -2000bp to +100bp relative to the TSS of CD52. A sorted list of non-redundant motifs ranked by their enrichment p-values were adjusted and displayed in supplemental Table S3. For hTFtarget analysis, the promoter sequence of human CD52 (-2000bp to +100bp relative to TSS) was uploaded (http://bioinfo.life.hust.edu.cn/hTFtarget#!/prediction) to predict candidate binding sites of STAT5. This prediction employs motifs curated from TRANSFAC/JASPAR/HOCOMOCO databases and ChIP-Seq datasets from hTFtarget portal. The potential STAT5 binding sites on CD52 promoter were displayed in supplemental Table S4.

Relative levels of *SETBP1* in CRISPRa-transduced MOLM-13 cells and AML patient samples

To compare the relative levels of *SETBP1* in CRISPRa-transduced MOLM-13 cells and AML patient samples, additional in-silico analysis and experiments were performed. Firstly, the transcription levels of *SETBP1* from AML cell lines (including MOLM-13) were retrieved from CCLE (Cancer Cell Line Encyclopedia) database (Public expression data, 2023Q2) using the DepMap portal (https://depmap.org/portal/) and present as TPM (transcripts per kilobase million). The transcription levels of *SETBP1* from AML patient samples were retrieved from public TCGA-LAML database, presented as TPM and analyzed using GEPIA2 program (http://gepia2.cancer-

pku.cn/#index). Secondly, the transcriptional levels of *SETBP1* in CRISPRa-transduced MOLM-13 cells and our achieved AML patient samples were detected by RT-qPCR and compared after normalizing to their corresponding GAPDH. Mononuclear cells (MNCs) from AML primary samples were isolated by Ficoll-Paque plus (Hyclone, Thermo Fisher Scientific) and preserved in liquid nitrogen until used.

Colony-formation assay

The colony-formation assay (CFU) of control and SETBP1-overexpressed MOLM-13 cells was performed using a standard methylcellulose-based system (MethoCult #4230, Stem Cell Technologies) as previously described.[8] Briefly, MOLM-13 cells were seeded in 24-well plates (100 cells/ well) in triplicates. Colonies were stained (Crystal Violet Staining Solution, Beyotime) and recorded by LEICA DMi1 microscopy after 10 days of standard culture.

Phagocytosis assay

THP-1 monocytic cells were transduced with lentivirus (LentiCRISPRv2-mCherry, Addgene # 99154, a gift from Agata Smogorzewska lab) encoding for mCherry and sorted by FACS (FACSaria FUSION Flow Cytometers, BD Biosciences). THP-1 cells were differentiated in macrophages as previously described.[15] Briefly, mCherry$^+$ THP-1 cells were seeded in a 24-wells plate (0.2 x 10^6/mL, 1mL) and treated with 20 ng/mL phorbol 12-myristate 13-acetate (PMA, MCE) in RPMI-1640 with 10% FBS for 48 hours. After differentiation, PMA was washed out with medium, and control or SETBP1-overexpressed (CTL vs OE) MOLM-13 target cells (EGFP$^+$) were added (E: T = 1: 2)

and co-cultured for 12 hours. Subsequently, nonadherent $EGFP^+$ MOLM-13 cells were washed out and the plate was imaged for mCherry and EGFP fluorescence (IX73 microscopy, Olympus). The phagocytic events were defined by double positive ($mCherry^+/EGFP^+$) signals and the average numbers were calculated based on three random fields of view per well. In separate experiments, flow cytometry was performed to quantify the percentage of phagocytic events after co-culture. After washing out the nonadherent $EGFP^+$ MOLM-13 cells (CTL vs OE), the adherent cells were harvested (0.25% Trypsin-EDTA, Thermo Fisher), washed by PBS twice, and subjected to flow cytometry (LSR Fortessa Cell Analyzer, BD Biosciences) analysis immediately. The phagocytic activities were quantified by the percentage of double positive ($mCherry^+/EGFP^+$) populations in each group. All experiments were performed in triplicate.

To generate primary macrophages, umbilical cord blood mononuclear cells (UCB-MNC) were differentiated with minor modifications as previously described.[16] Briefly, UCB-MNCs were isolated using Ficoll-Paque Plus (HyClone, Thermo Fisher Scientific), washed twice in PBS, and resuspended in RPMI-1640 with 10% FBS. MNCs were then seeded in a 24-well plate (1 x 10^6/mL, 1mL) and allowed to adhere for 2 hours at 37℃. The adherent monocytes were cultured in RPMI-1640 supplemented with 10% FBS and 10 ng/mL recombinant human granulocyte-macrophage colony-stimulating factor (GM-CSF, PeproTech) for 7 days. Differentiated macrophages were washed with PBS twice and stained with CM-Dil (1 μmol/mL, Yeasen). The EGFP-positive

control or SETBP1-overexpressed MOLM-13 cells (CTL vs OE) were then added and co-culture for 6 hours. The phagocytic activities were recorded based on fluorescent imaging (CM-Dil and EGFP) and the phagocytosis events per field were calculated as shown above.

Gene Set Enrichment Analysis and immune cells infiltrations

Gene Set Enrichment Analysis and immune cell infiltrations were performed as our previously described.[14] Briefly, the *SETBP1*-correlated genes ($p < 0.05$) were shortlisted and retrieved from RNA-seq data from the TCGA-LAML database using the "LinkFinder" section of the LinkedOmics program. *SETBP1*-correlated genes were subjected to Gene Set Enrichment Analysis (GSEA) analysis using the "LinkInterpreter" section of the LinkedOmics program with default setting (Minimum Number of Genes = 3; Simulations = 500; GO analysis of biological process). Enrichment results were displayed as "Normalized Enrichment Score" of significant GO terms (FDR < 0.05). The correlation between *SETBP1* expression and immune cell infiltrations in human AML patients (TCGA-LAML database) was analyzed using the QUANTISEQ algorithm by the SangerBox program.

Ethics approval and statement

All animal studies have been approved by the Ethical Committee at The Fifth Affiliated Hospital of Sun Yat-sen University. Informed consent was obtained from all subjects and the human studies were approved by the Institutional Review Boards from The Fifth Affiliated Hospital of Sun Yat-sen University. All experiments conformed to the principles set out in the WMA Declaration of Helsinki and the Department of Health and Human Services Belmont Report.

Statistical analysis

The sample size used in this study was chosen based on the pilot study and specified in each section. Zebrafish embryos at defined developmental stages or ages were randomly allocated for each experiment. All experiments were performed in triplicates and data were presented as mean ± standard error of the mean (SEM) unless otherwise specified. Results were compared with the student's t-test / Mann-Whitney U test (numerical data) or Chi-Square test / Fisher's exact test (categorical data). Survival data was analyzed using the Kaplan-Meier method (log-rank test). All statistical analyses were performed using GraphPad Prism 8.0.1. P-values less than 0.05 were considered statistically significant unless specified.

Reference

1. Fagerberg, L. et al. Analysis of the human tissue-specific expression by genome-wide integration of transcriptomics and antibody-based proteomics. *Mol Cell Proteomics* **13**, 397-406 (2014).
2. Bagger, F.O., Kinalis, S. & Rapin, N. BloodSpot: a database of healthy and malignant haematopoiesis updated with purified and single cell mRNA sequencing profiles. *Nucleic Acids Res* **47**, D881-D885 (2019).
3. Cerami, E. et al. The cBio cancer genomics portal: an open platform for exploring multidimensional cancer genomics data. *Cancer Discov* **2**, 401-404 (2012).
4. Vasaikar, S.V., Straub, P., Wang, J. & Zhang, B. LinkedOmics: analyzing multi-omics data within and across 32 cancer types. *Nucleic Acids Res* **46**, D956-D963 (2018).
5. Heinz, S. et al. Simple combinations of lineage-determining transcription factors prime cis-regulatory elements required for macrophage and B cell identities. *Mol Cell* **38**, 576-589 (2010).
6. Zhang, Q. et al. hTFtarget: A Comprehensive Database for Regulations of Human Transcription Factors and Their Targets. *Genomics Proteomics Bioinformatics* **18**, 120-128 (2020).
7. Kimmel, C.B., Ballard, W.W., Kimmel, S.R., Ullmann, B. & Schilling, T.F. Stages of embryonic development of the zebrafish. *Dev Dyn* **203**, 253-310 (1995).
8. He, B.L. et al. Follistatin is a novel therapeutic target and biomarker in FLT3/ITD acute myeloid leukemia. *EMBO Mol Med* **12**, e10895 (2020).
9. He, B.L. et al. Functions of flt3 in zebrafish hematopoiesis and its relevance to human acute myeloid leukemia. *Blood* **123**, 2518-2529 (2014).
10. Warkentin, A.A. et al. Overcoming myelosuppression due to synthetic lethal toxicity for FLT3-targeted acute myeloid leukemia therapy. *Elife* **3** (2014).
11. Ma, A.C.H., Shi, X., He, B.L., Guo, Y. & Leung, A.Y.H. A Zebrafish Model for Evaluating the Function of Human Leukemic Gene IDH1 and Its Mutation. *Methods Mol Biol* **1633**, 193-218 (2017).
12. Lam, S.S. et al. Homoharringtonine (omacetaxine mepesuccinate) as an adjunct for FLT3-ITD acute myeloid leukemia. *Sci Transl Med* **8**, 359ra129 (2016).
13. Konermann, S. et al. Genome-scale transcriptional activation by an engineered CRISPR-Cas9 complex. *Nature* **517**, 583-588 (2015).
14. Lai, W.J. et al. Pivotal role of cytosolic phospholipase PLA2G4A in the pathogenesis of FLT3-ITD-mutated acute myeloid leukemia. *Genes Dis* **10**, 22-25 (2023).
15. Schwende, H., Fitzke, E., Ambs, P. & Dieter, P. Differences in the state of differentiation of THP-1 cells induced by phorbol ester and 1,25-dihydroxyvitamin D3. *J Leukoc Biol* **59**, 555-561 (1996).

16. Wu, Z.H. et al. Preclinical characterization of the novel anti-SIRPalpha antibody BR105 that targets the myeloid immune checkpoint. *J Immunother Cancer* **10** (2022).

Supplemental Figures, Legends and Tables

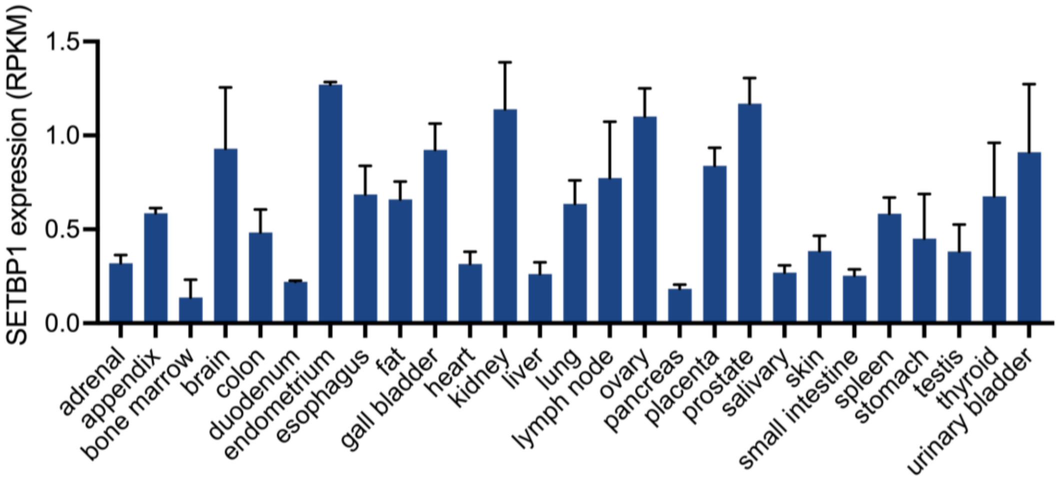

Figure S1. Relative expression of human *SETBP1* from different normal tissues in human. The relative expression of human *SETBP1* in human normal tissues were analyzed based on the HPA RNA-seq data and retrieved from NCBI gene portal (Gene ID: 26040). RPKM, reads per kilobase per million reads placed.

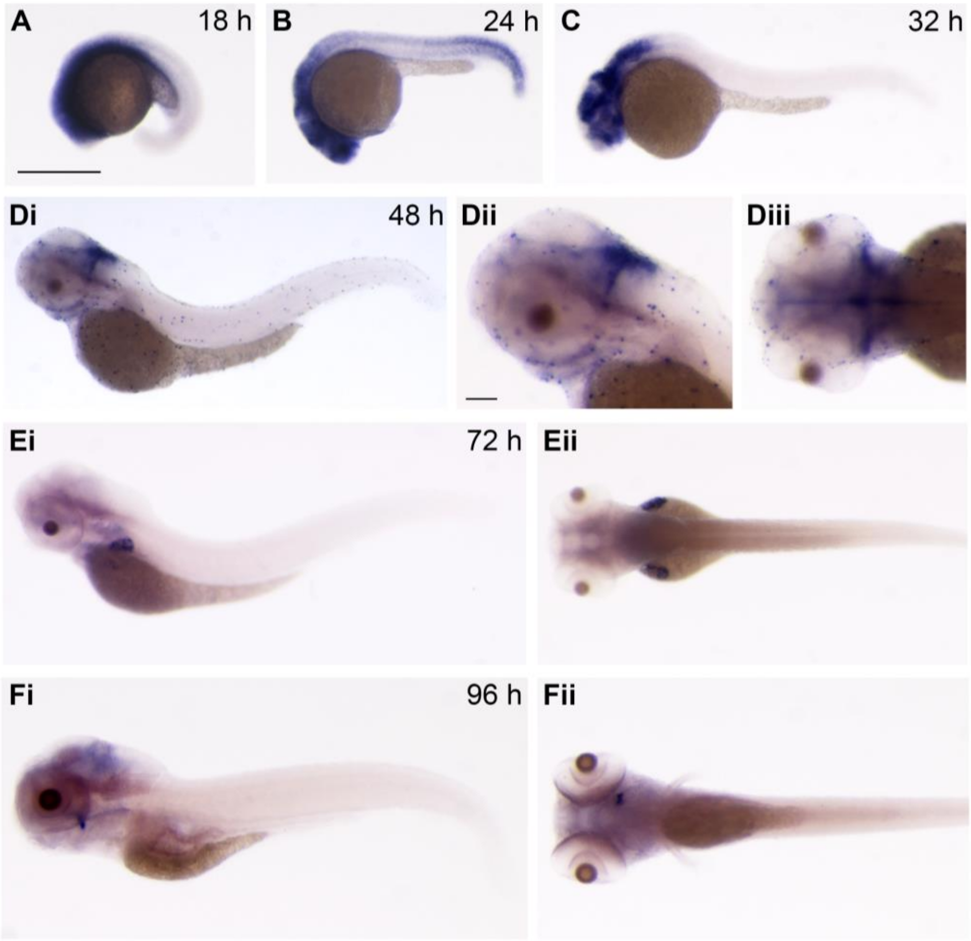

Figure S2. Detection of *setbp1* transcription by whole-mount in situ hybridization assay in zebrafish embryos. (A-F) Whole-mount in situ hybridization (WISH) assay detecting the transcription of zebrafish *setbp1* at 18 (A), 24 (B), 32 (C), 48 (Di-iii), 72 (Ei-ii), and 96 (Fi-ii) hpf. Scale bar = 500 µm (A-F) except for panels Dii and Diii (100 µm).

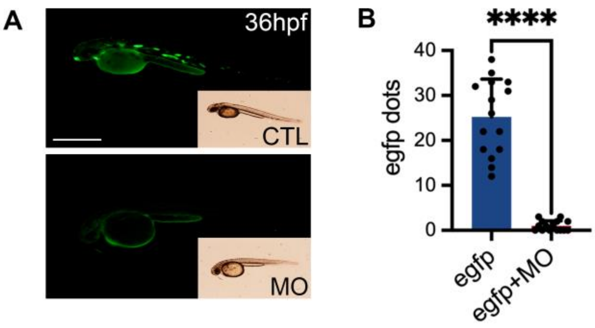

Figure S3. Knockdown efficiency of *setbp1* morpholino in zebrafish embryos. (A) *setbp1UTR-egfp* chimeric gene was co-injected with control MO or setbp1 MO into one-cell stage embryos. The *setbp1UTR-egfp* chimeric gene contains the setbp1 MO binding site. (B) Quantification of egfp$^+$ cells of embryos (CTL vs MO) at 36hpf. Scale bar = 500 µm.

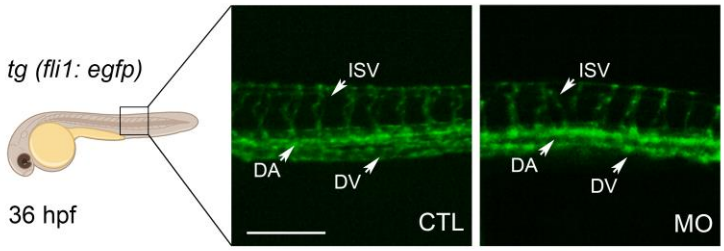

Figure S4. Effects of *setbp1* knockdown on embryonic vasculogenesis and angiogenesis. Control MO (CTL) or setbp1 MO (MO) was microinjected into one-cell stage embryos from *Tg(fli1: egfp)* transgenic reporter line. The development of dorsal aorta (DA), dorsal vein (DV), and intersegmental vessels (ISVs) are intact in both groups. Scale bar = 100 µm.

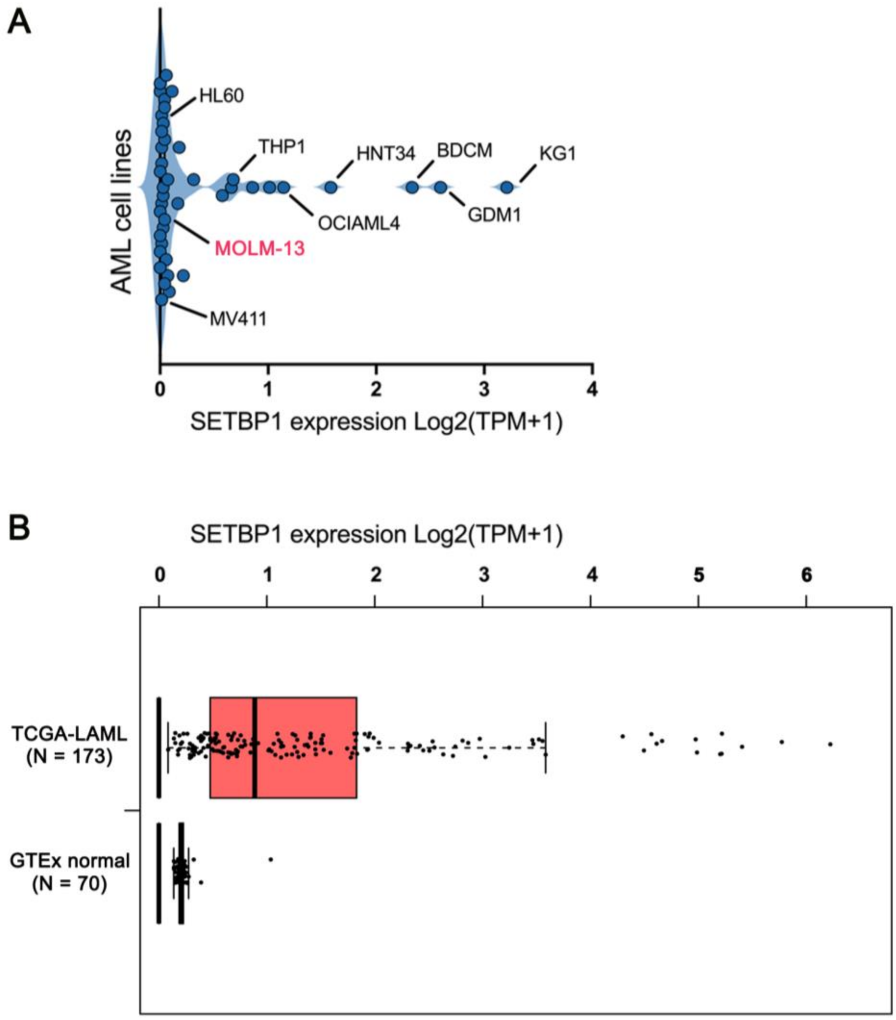

Figure S5. Relative transcription level of *SETBP1* in different AML cells lines and AML patient samples. (A) The relative transcription levels of *SETBP1* in AML cell lines were analyzed using the "TPM" dataset from Cancer Cell Line Encyclopedia (CCLE) portal. The data from AML cell lines were shortlisted and the relative transcription levels of *SETBP1* were analyzed. TPM, transcript per million. **(B)** The expression of *SETBP1* in AML patient samples (TCGA-LAML database) and normal control from The Genotype-Tissue Expression (GTEx) project was retrieved and analyzed using GEPIA2 program.

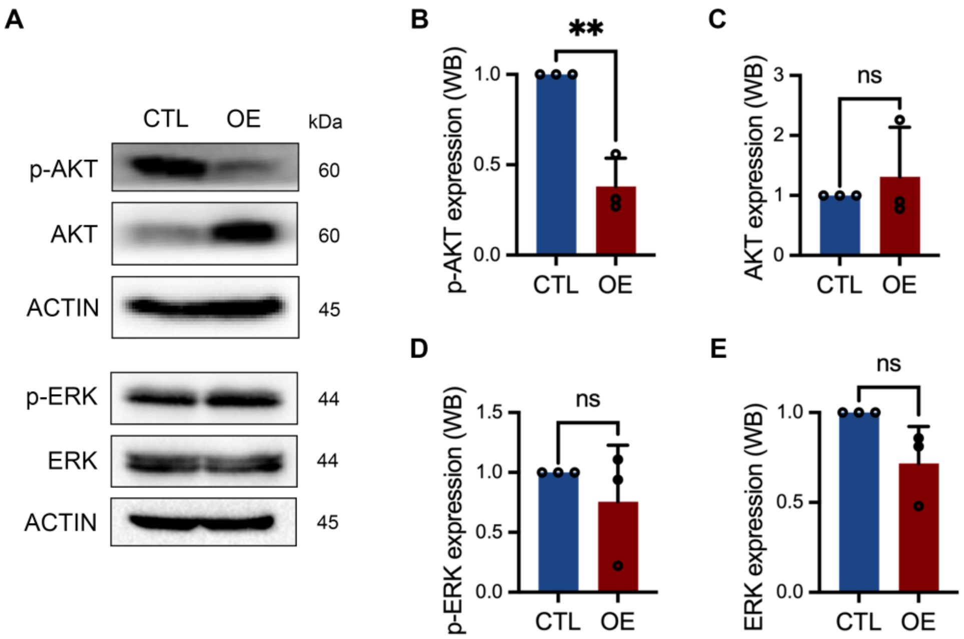

Figure S6. Western Blotting detecting FLT3 signal pathway in *SETBP1-*overexpressed MOLM-13 cells. (A-E) Western Blotting was performed to detect the expression and phosphorylation of AKT and ERK in control (CTL) and SETBP1 overexpressed (OE) MOLM-13 cells (A). The intensity of bands was quantified by ImageJ software and the relative expression was calculated after normalizing to the corresponding β-ACTIN (B-E).

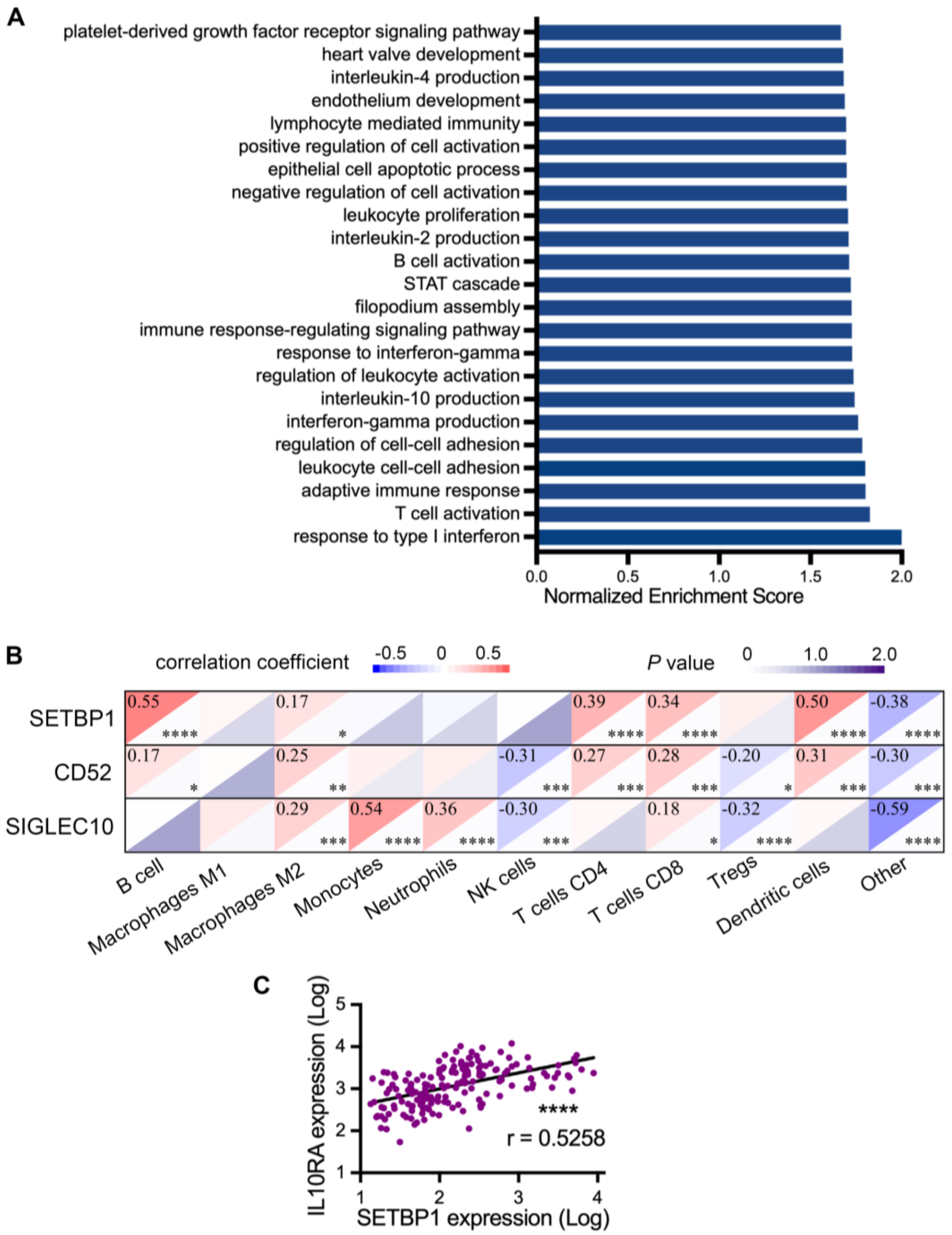

Figure S7. SETBP1-associated genetic and immune signatures in AML. (A) SETBP1-associated genes were shortlisted by the LinkedOmics program and subjected to GSEA analysis with default settings. (B) Correlation between gene expression and immune cell infiltrations by SangBox program using QUANTISEQ algorithm. (C) The correlation between *SETBP1* and *IL10RA* expression was analyzed by the LinkedOmics program using the TCGA-LAML database.

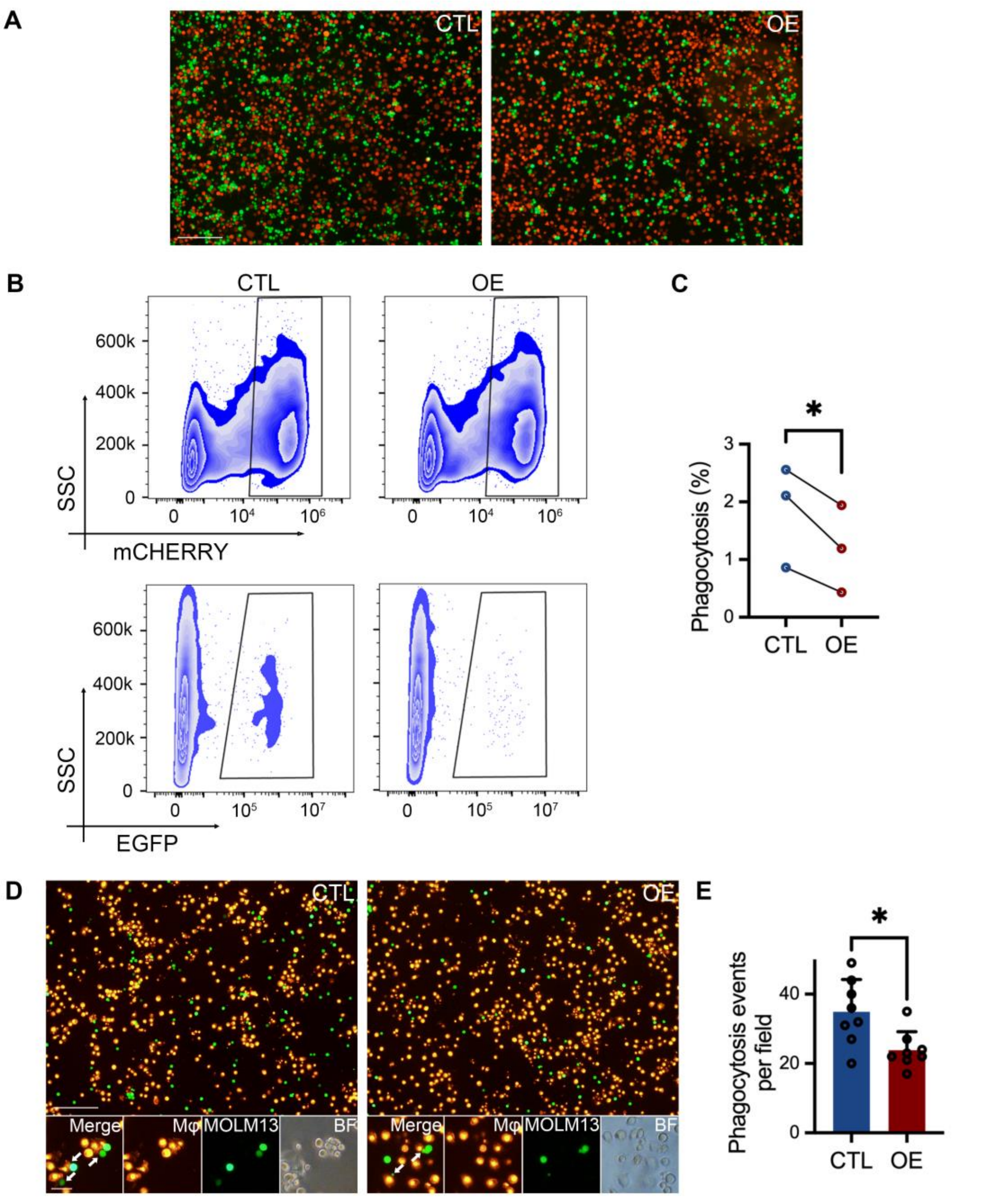

Figure S8. Co-culture and phagocytosis assay. Control (CTL) and SETBP1-overexpressed (OE) MOLM-13 cells (EGFP^{+}) were co-cultured with mCherry^{+} THP-1-derived macrophages (A) or CM-Dil-labelled UCB-MNCs-derived primary macrophages (D). Percentages of EGFP^{+} cells within the mCherry^{+} population were detected by flow cytometry (B-C). Phagocytosis events per field were recorded by fluorescent imaging (D-E). Scale bar = 200 µm (A); 200 µm (D, upper panel); and 10 µm (D, lower panel).

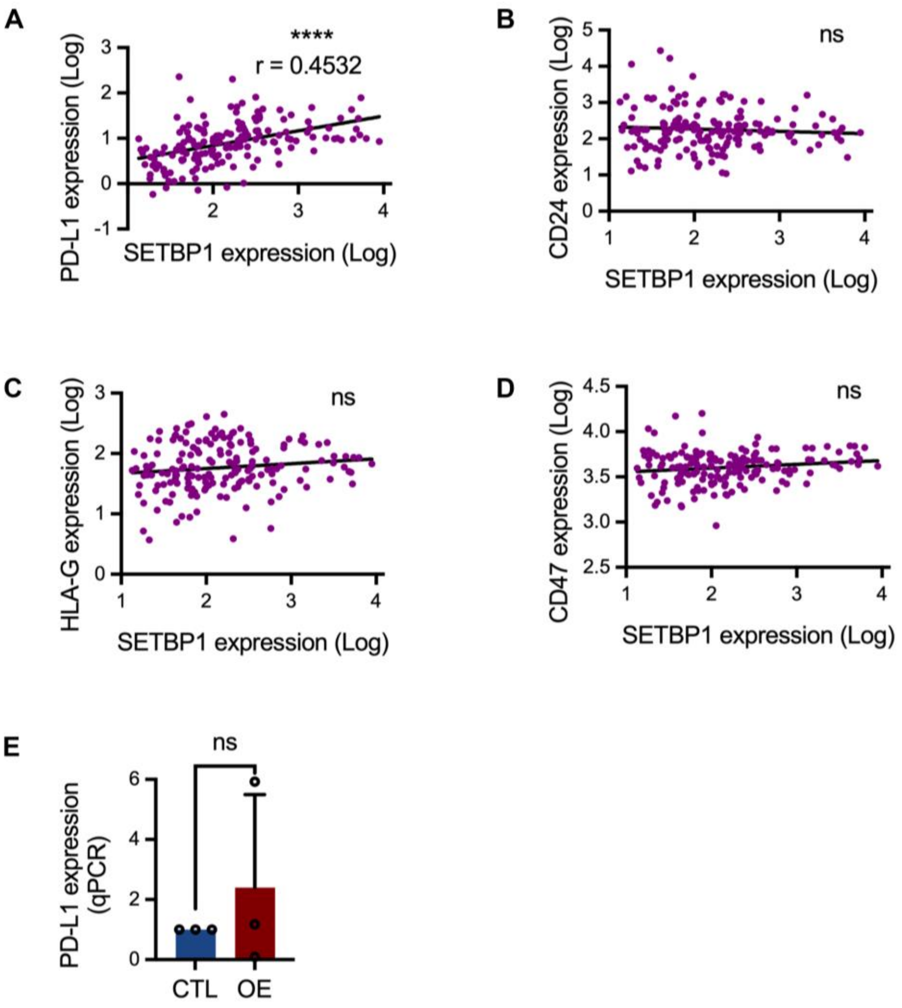

Figure S9. Correlation between SETBP1 and phagocytic checkpoints in AML. (A-D) Correlation between *SETBP1* and phagocytic checkpoints (*PD-L1*, *CD24*, *HLA-G,* and *CD47*) was analyzed by the LinkedOmics program (Pearson correlation test) using the TCGA-LAML database. (E) RT-qPCR assay detecting the expression of *PD-L1* in control (CTL) and SETBP1-overexpressed (OE) MOLM-13 cells.

Table S1. Summary of primers and oligos used in this study.

Name	Sequence (5'-3')
**Morpholino-mediated gene knockdown**	
setbp1 MO	TCTTTGCTCCATTGACGACCCTGAA
CTL MO	CCTCTTACCTCAGTTACAATTTATA
**Real-time quantitative PCR**	
GAPDH-qPCR-fwd	AGGTCGGTGTGAACGGATTTG
GAPDH-qPCR-rev	GGGGTCGTTGATGGCAACA
SETBP1-qPCR-fwd	AAACCCGGTTCTCTCACTCT
SETBP1-qPCR-rev	AAGAACAGGGAATGACCACAGG
PD-L1-qPCR-fwd	GGACAAGCAGTGACCATCAAG
PD-L1-qPCR-rev	CCCAGAATTACCAAGTGAGTCCT
CD52-qPCR-fwd	TCTTCCTCCTACTCACCATCAG
CD52-qPCR-rev	CCTCCGCTTATGTTGCTGGA
**Whole-mount in situ hybridization**	
setbp1-ISH-fwd	ACTGAGAAAGGCCCAGCATC
setbp1-ISH-rev	GCTTGCCTCTTTGCTTGTCC
**CRISPRa-mediated gene overexpression**	
SETBP1 sgRNA	AACCCGCCAGCCCCGCCAGC
Scramble sgRNA	CTGAAAAAGGAAGGAGTTGA

Table S2. Reagents and resources used in this study.

Name	Source	Identifier
**Primary antibody for Western Blotting**		
Anti SETBP1 antibody (1:500 dilution)	Abcam	ab98222
Anti FLT3 (8F2) antibody (1:1000 dilution)	CST	3462S
Anti phospho-FLT3 (Tyr591) antibody (1:500 dilution)	CST	3461S
Anti STAT5 (D3N2B) antibody (1:1000 dilution)	CST	25656S
Anti phospho-STAT5 (Tyr694) antibody (1:1000 dilution)	CST	9351S
Anti AKT antibody (1:1000 dilution)	CST	4685S
Anti phospho-AKT (Ser473) antibody (1:1000 dilution)	CST	9271S
Anti p44/42 (ERK1/2) antibody (1:1000 dilution)	CST	9102S
Anti phospho-p44/42 (ERK1/2) antibody (1:1000 dilution)	CST	4370S
Anti β-ACTIN (1:5000 dilution)	Sigma	A2228
**Software**		
Flowjo	FLOWJO LLC	Version: 7.6.1
Prism	GraphPad	Version: 6.0c
ImageJ	NIH	Version: 1.53k
Photoshop	Adobe	Version: 2017.0.0

Table S3. Transcriptional levels of SETBP1 in AML cell lines from CCLE database.

Name	Cell types	DepMap ID	Log_2(TPM+1)
KG1	Acute Myeloid Leukemia	ACH-000386	3.21
GDM1	Acute Myeloid Leukemia	ACH-000081	2.60
BDCM	Acute Myeloid Leukemia	ACH-000080	2.33
HNT34	Acute Myeloid Leukemia	ACH-000299	1.58
OCIAML4	Acute Myeloid Leukemia	ACH-001613	1.14
HDMYZ	Acute Myeloid Leukemia	ACH-000190	1.01
MUTZ3	Acute Myeloid Leukemia	ACH-000084	0.86
SKNO1	Acute Myeloid Leukemia	ACH-001656	0.68
THP1	Acute Myeloid Leukemia	ACH-000146	0.66
OCIAML5	Acute Myeloid Leukemia	ACH-000065	0.58
SKM1	Acute Myeloid Leukemia	ACH-000373	0.31
TF1	Acute Myeloid Leukemia	ACH-000387	0.21
KO52	Acute Myeloid Leukemia	ACH-000498	0.18
P31FUJ	Acute Myeloid Leukemia	ACH-000770	0.16
AML193	Acute Myeloid Leukemia	ACH-000557	0.11
MUTZ8	Acute Myeloid Leukemia	ACH-001577	0.08
OCIM1	Acute Myeloid Leukemia	ACH-000751	0.07
NB4	Acute Myeloid Leukemia	ACH-000294	0.07
PL21	Acute Myeloid Leukemia	ACH-000218	0.06
NOMO1	Acute Myeloid Leukemia	ACH-000168	0.06
U937	Acute Myeloid Leukemia	ACH-000406	0.04
KASUMI6	Acute Myeloid Leukemia	ACH-000166	0.04
KASUMI1	Acute Myeloid Leukemia	ACH-000263	0.04
**MOLM13**	**Acute Myeloid Leukemia**	**ACH-000362**	**0.04**
OCIAML2	Acute Myeloid Leukemia	ACH-000113	0.04
MONOMAC6	Acute Myeloid Leukemia	ACH-000006	0.03
MONOMAC1	Acute Myeloid Leukemia	ACH-001129	0.03
ME1	Acute Myeloid Leukemia	ACH-000439	0.03
SET2	Acute Myeloid Leukemia	ACH-000195	0.03
SIGM5	Acute Myeloid Leukemia	ACH-000112	0.01
HL60	Acute Myeloid Leukemia	ACH-000002	0.01
CMK	Acute Myeloid Leukemia	ACH-000641	0.01
OCIAML3	Acute Myeloid Leukemia	ACH-000336	0.01
**MV411**	**Acute Myeloid Leukemia**	**ACH-000045**	**0.01**
M07E	Acute Myeloid Leukemia	ACH-000602	0.01
HEL9217	Acute Myeloid Leukemia	ACH-000005	0.01
PLB985	Acute Myeloid Leukemia	ACH-000034	0.00
OCIM2	Acute Myeloid Leukemia	ACH-001618	0.00
SHI1	Acute Myeloid Leukemia	ACH-001647	0.00
MOLM16	Acute Myeloid Leukemia	ACH-000369	0.00
HEL	Acute Myeloid Leukemia	ACH-000004	0.00
CMK115	Acute Myeloid Leukemia	ACH-001036	0.00
MOLM14	Acute Myeloid Leukemia	ACH-001574	0.00

Note: MOLM13 and MV411, FLT3-ITD-mutated AML cell lines.

Table S4. Prediction of transcription factor binding motifs in CD52 promoter by HOMER analysis.

Motif	Match TF name/ID	Source	Strand	P value
**STAT5**				
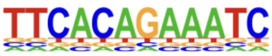	Stat5b/MA1625.1	Jaspar	+	1.00E-04
	Stat5(Stat)/ChIP-Seq	GSE12346	-	1.00E-04
	Stat5a/MA0519.1	Jaspar	+	1.00E-04
**Other TFs**				
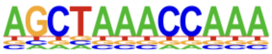	Irf6_1/PB0036.1	Jaspar	+	1.00E-04
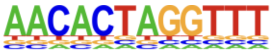	Sox15_1/PB0065.1	Jaspar	+	1.00E-04
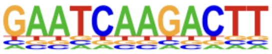	ONECUT1/MA0679.2	Jaspar	+	1.00E-04
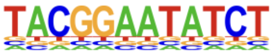	DPRX/MA1480.1	Jaspar	-	1.00E-04
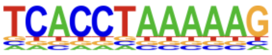	HOXC13/MA0907.1	Jaspar	+	1.00E-04
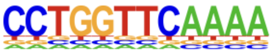	CHR(?)/Hela-CellCycle-Expression	Homer	-	1.00E-04
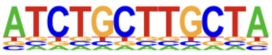	ETS:E-box/ChIP-Seq	GSE22178	-	1.00E-04
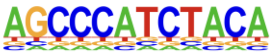	ZNF449/MA1656.1	Jaspar	+	1.00E-04
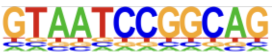	OTX1/MA0711.1	Jaspar	+	1.00E-04
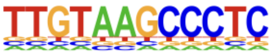	Pitx1(Homeobox)	GSE38910	+	1.00E-04
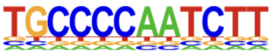	YY1(Zf)/Promoter	Homer	-	1.00E-04
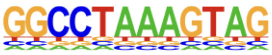	Mef2a(MADS)/ChIP-Seq	GSE21529	+	1.00E-04
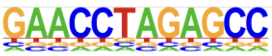	ZBTB6/MA1581.1	Jaspar	+	1.00E-04
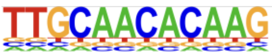	CEBP:AP1(bZIP)/ChIP-Seq	GSE21512	-	1.00E-04
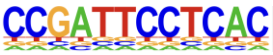	RELB/MA1117.1	Jaspar	+	1.00E-04

ACAATAGAAGAG	Sry_2/PB0183.1	Jaspar	+	1.00E-04
TGTAGCTTAGTC	BACH1/MA1633.1	Jaspar	-	1.00E-04
AGATCTCCCCTG	EBF1/MA0154.4	Jaspar	-	1.00E-04
CCCACTTGTCCC	NKX2-3/MA0672.1	Jaspar	+	1.00E-04
CAAAATTCTAGG	ZNF136/MA1588.1	Jaspar	+	1.00E-04
ACGATCTAGCCT	Zbtb12_1/PB0090.1	Jaspar	+	1.00E-04
CAGAGGTTACAT	DMRTA2/MA1478.1	Jaspar	+	1.00E-04
TTCCCTTTACTA	HMBOX1/MA0895.1	Jaspar	-	1.00E-04
AACTGAAACCAG	IRF4(IRF)/ChIP-Seq	GSE32465	+	1.00E-04
GTTGTGGTGACC	RUNX-AML(Runt)/ChIP-Seq(Barski_et_al.)	Homer	+	1.00E-04
CAACCCTTCCAC	ZNF467(Zf)/ChIP-Seq	GSE58341	-	1.00E-04
CAGCTCCTATAC	Ascl2/MA0816.1	Jaspar	+	1.00E-04
GCCACGAAGATC	Npas4(bHLH)/ChIP-Seq	GSE127793	+	1.00E-04
CTACCAAAATCA	Osr1_2/PB0154.1	Jaspar	+	1.00E-04
CTCACCCCGCCT	Ascl2_2/PB0107.1	Jaspar	+	1.00E-04
TAAATTGGTTAT	Msx3/PH0108.1	Jaspar	-	1.00E-04
GGTATTATCCAC	ZNF354C/MA0130.1	Jaspar	+	1.00E-04
CCTAGCATGTAA	Zic2_2/PB0206.1	Jaspar	+	1.00E-04
GCTACCAAGACA	Osr1_2/PB0154.1	Jaspar	+	1.00E-04
GGATTGACCTCT	COUP-TFII(NR)/ChIP-Seq	GSE46497	-	1.00E-04
TTCCCTTGAAAC	STAT6(Stat)/ChIP-Seq	GSE38377	+	1.00E-04
TATGTTCTGGCT	Foxj3_2/PB0121.1	Jaspar	-	1.00E-04

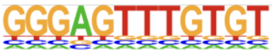	Myb_2/PB0149.1	Jaspar	-	1.00E-04
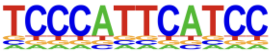	Sox17_2/PB0170.1	Jaspar	+	1.00E-04
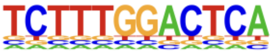	HNF4A(var.2)/MA1494.1	Jaspar	-	1.00E-04
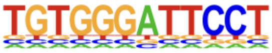	ZNF75D/MA1601.1	Jaspar	+	1.00E-04
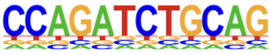	ZBTB18/MA0698.1	Jaspar	+	1.00E-04
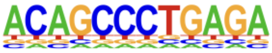	ZNF341/MA1655.1	Jaspar	+	1.00E-04
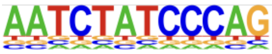	Zfp410_1/PB0098.1	Jaspar	-	1.00E-04
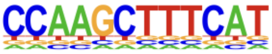	Nr2e3/MA0164.1	Jaspar	+	1.00E-04
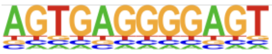	MAZ/MA1522.1	Jaspar	-	1.00E-04
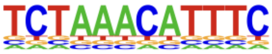	Foxq1/MA0040.1	Jaspar	-	1.00E-03
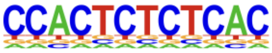	Zfp281(Zf)/ChIP-Seq	GSE81042	+	1.00E-03
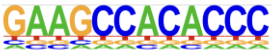	EKLF(Zf)/ChIP-Seq	GSE20478	-	1.00E-03

Table S5. Prediction of STAT5 binding sites in CD52 promoter by hTFtarget analysis.

Source	Site	Strand	Score	P value	Matched motif
**STAT5A** 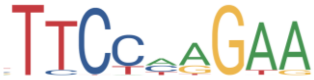					
hTFtarget[1]	-645 ~ -634	+	14.37	8.75E-06	TTCCTTGAAATG
hTFtarget	-676 ~ -661	+	10.60	1.25E-05	CTTCAGAAATAATCTA
hTFtarget	67 ~ 75	+	13.04	1.61E-05	CGCTTCCTC
hTFtarget	-647 ~ -636	-	12.93	2.36E-05	TTTCAAGGAACT
hTFtarget	-648 ~ -637	-	12.65	2.37E-05	TTCAAGGAACTG
database[2]	-565 ~ -553	-	12.65	2.60E-05	GGATTTCTGTGAA
hTFtarget	-653 ~ -638	+	6.17	3.07E-05	CTTCCCAGTTCCTTGA
database	-645 ~ -633	-	12.08	3.62E-05	TCATTTCAAGGAA
hTFtarget	-1154 ~ -1143	+	11.34	4.80E-05	CACTCTCTCACC
hTFtarget	-848 ~ -842	+	13.39	5.35E-05	CACACAC
database	-647 ~ -636	+	11.54	5.37E-05	AGTTCCTTGAAA
hTFtarget	-1085 ~ -1074	+	11.11	5.42E-05	CATTCATCCTCA
hTFtarget	-1550 ~ -1539	-	9.69	5.59E-05	CACACTCTCCCT
hTFtarget	73 ~ 81	+	11.15	8.64E-05	CTCTTCCTC
hTFtarget	-566 ~ -555	+	10.86	8.97E-05	CTTCACAGAAAT
hTFtarget	-565 ~ -554	+	9.68	9.06E-05	TTCACAGAAATC
hTFtarget	-1180 ~ -1165	+	2.59	9.08E-05	CCCACCCCTCCCTGGG
**STAT5B** 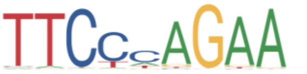					
database	-647 ~ -635	-	13.00	1.87E-05	ATTTCAAGGAACT
hTFtarget	-1395 ~ -1387	-	13.42	2.05E-05	AAGCCACAG
hTFtarget	-647 ~ -632	+	12.54	3.15E-05	AGTTCCTTGAAATGAG
hTFtarget	-793 ~ -778	-	9.97	3.26E-05	AACCTCCACCTCCTGG
hTFtarget	-170 ~ -155	+	9.96	3.28E-05	CTGCTCCCCCAGCCAG
hTFtarget	-650 ~ -635	-	12.16	4.08E-05	ATTTCAAGGAACTGGG
hTFtarget	-567 ~ -552	+	11.68	5.56E-05	CCTTCACAGAAATCCA
hTFtarget	-593 ~ -585	+	10.72	8.50E-05	GGCTTCCCG

[1] *hTFtarget: ChIP-Seq datasets from hTFtarget*

[2] *Database: TRANSFAC/JASPAR/HOCOMOCO databases*
